# Ability of *Lactobacillus fermentum *to overcome host α-galactosidase deficiency, as evidenced by reduction of hydrogen excretion in rats consuming soya α-galacto-oligosaccharides

**DOI:** 10.1186/1471-2180-8-22

**Published:** 2008-01-29

**Authors:** Jean Guy LeBlanc, Florence Ledue-Clier, Martine Bensaada, Graciela Savoy de Giori, Theodora Guerekobaya, Fernando Sesma, Vincent Juillard, Sylvie Rabot, Jean-Christophe Piard

**Affiliations:** 1 Centro de Referencia para Lactobacilos (CERELA-CONICET), Chacabuco 145, San Miguel de Tucumán, Tucumán, T4000ILC, Argentina; 2 INRA, UR 888 – Lactic Acid Bacteria and Opportunistic Pathogens (UBLO), F-78350 Jouy-en-Josas, France; 3 INRA, UR 910 – Ecology and Physiology of the Digestive Tract (UEPSD), F-78350 Jouy-en-Josas, France; 4 Cátedra de Microbiología Superior, Universidad Nacional de Tucumán (UNT), San Miguel de Tucumán, Tucumán, T4000ILC, Argentina

## Abstract

**Background:**

Soya and its derivatives represent nutritionally high quality food products whose major drawback is their high content of α-galacto-oligosaccharides. These are not digested in the small intestine due to the natural absence of tissular α-galactosidase in mammals. The passage of these carbohydrates to the large intestine makes them available for fermentation by gas-producing bacteria leading to intestinal flatulence. The aim of the work reported here was to assess the ability of α-galactosidase-producing lactobacilli to improve the digestibility of α-galacto-oligosaccharides *in situ*.

**Results:**

Gnotobiotic rats were orally fed with soy milk and placed in respiratory chambers designed to monitor fermentative gas excretion. The validity of the animal model was first checked using gnotobiotic rats monoassociated with a *Clostridium butyricum *hydrogen (H_2_)-producing strain. Ingestion of native soy milk by these rats caused significant H_2 _emission while ingestion of α-galacto-oligosaccharide-free soy milk did not, thus validating the experimental system. When native soy milk was fermented using the α-galactosidase-producing *Lactobacillus fermentum *CRL722 strain, the resulting product failed to induce H_2 _emission in rats thus validating the bacterial model. When *L. fermentum *CRL722 was coadministered with native soy milk, a significant reduction (50 %, *P *= 0.019) in H_2 _emission was observed, showing that α-galactosidase from *L. fermentum *CRL722 remained active *in situ*, in the gastrointestinal tract of rats monoassociated with *C. butyricum*. In human-microbiota associated rats, *L. fermentum *CRL722 also induced a significant reduction of H_2 _emission (70 %, *P *= 0.004).

**Conclusion:**

These results strongly suggest that *L. fermentum *α-galactosidase is able to partially alleviate α-galactosidase deficiency in rats. This offers interesting perspectives in various applications in which lactic acid bacteria could be used as a vector for delivery of digestive enzymes in man and animals.

## Background

The nutritional value of soya-derived products is high since the amino acid profile of soy protein corresponds more closely to human requirements than most other plant proteins [1]. In recognition of this high quality, the US Department of Agriculture issued a ruling in 2000 allowing soy protein to completely replace animal protein in the Federal School Lunch Program [2]. Soy proteins have also been shown to reduce the incidence of cardiovascular disease [3] and, in this context, the US Food and Drug Administration has approved a health claim for the cholesterol-lowering effect of soy proteins [4]. Other key benefits of soya derive from its high content of isoflavones which are thought to exert a range of biological effects against hormone-dependent diseases such as breast and prostate cancer, menopausal symptoms, cardiovascular diseases, and osteoporosis [5]. Taken together, these reported health benefits, along with growing consumer preference for plant-derived food rather than meat, have led to an increasing demand for soy products [6]. However, in addition to these positive properties, soya does have one negative characteristic limiting its use in human nutrition. As is the case with other legumes, soya contains high levels of the α-galacto-oligosaccharides (α-GOS) raffinose and stachyose that are composed of one sucrose moiety and one or two galactose moieties, respectively. Since mammals are deficient in the enzyme α-galactosidase (α-Gal), which hydrolyses the α-1, 6 linkages found in these sugars, α-GOS are not digested in the upper gastrointestinal tract and reach the large intestine where they are fermented by the resident microbiota. The resulting production of fermentative gases can induce abdominal pain as well as the social embarrassment associated with flatulence [7,8]. Such negative aspects reduce the acceptability of soy products as a major human food source [9].

One means of avoiding this problem could be to remove α-GOS from the raw agricultural products. This could be achieved by fermenting soya-derived products, such as soy milk, using food grade bacteria that are able to catabolize α-GOS into digestible carbohydrates. Lactic acid bacteria (LAB) have long been used in food processing, and some of these are able to produce α-Gal, making them good candidates to fulfil this task [10-12]. This approach is promising for the manufacture of soy yoghurts since some LAB are also able to reduce the concentration of the aldehydes responsible for the undesirable beany flavour of soy milk [13]. However, this strategy is limited to food products that are to be fermented by LAB. For soya and α-GOS-rich food products that are not intended to be fermented by LAB, another method of reducing the quantity of gas-producing substrates would be to use LAB as a vehicle for delivering α-Gal to the small intestine, allowing the enzyme to hydrolyse undigested α-GOS prior to their passage to the large intestine. In previous experiments, we selected the *Lactobacillus *(*L*.) *fermentum *CRL722 strain for its high α-Gal activity, which allowed it to degrade raffinose and stachyose *in vitro *[11,14]. Following oral administration of live cells or cell-free extracts of this strain, we were able to detect a short-lived α-Gal activity in the small intestinal chyme of conventional rats [15].

The present study was designed to assess the efficiency of *L. fermentum *CRL722 to reduce gas production in rats consuming α-GOS. Gnotobiotic rats which were monoassociated with *Clostridium *(*C*.)* butyricum*, a bacterial species known to produce large amounts of gas from carbohydrate fermentation, were first used as an animal model. In a second series of experiments, we investigated the effect of *L. fermentum *CRL722 in a microbiological environment similar to the human gastrointestinal tract, using human microbiota-associated rats. In all the trials, *L. fermentum *CRL722 was consumed as a bolus of live cells, and H_2 _excreted through breath and flatus was used as a biomarker of gas production in the gastrointestinal tract.

## Results

### A model experimental system to assess the potential of α-Gal-producing lactobacilli to improve α-GOS digestion in rats

**Figure 1:**
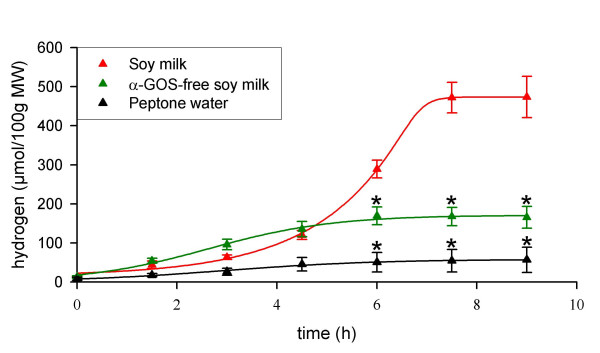
**α-GOS-induced H_2 _excretion in gnotobiotic rats monoassociated with *C. butyricum *DSM10702 (Cb rats)**. The rats (*n *= 12) were administered intragastrically with the solution to be tested and they were placed in respiratory chambers. Air samples taken from the chambers were analyzed for H_2 _concentration using GC. Treatments successively applied to all rats (for more details, see Material and Methods section and Fig. 5) are boxed. Cross bars indicate standard error of the mean. MW, metabolic weight; *, indicates that *P *values (Student-Newman-Keuls test) of the differences with the group receiving soy milk is *P* < 0.01.

A model system was designed in order to assess the potential of *L. fermentum*-delivered α-Gal to improve the digestion of α-GOS in rats. This system made use of respiratory chambers to monitor gas excretion in gnotobiotic rats in which the microbiota was first simplified to a single strain of *C. butyricum *that produces H_2 _from α-GOS fermentation (Cb rats). In these rats, the colonization of the gastrointestinal tract by *C. butyricum *ranged from 10^7 ^to 10^8 ^CFU/g of faeces. Initial experiments showed that administration of 1 ml of native soy milk with raffinose and stachyose levels of 4.0 and 7.5 mM, respectively, did not significantly induce H_2 _emission in Cb rats (data not shown). Therefore, native soy milk was enriched with various amounts of both α-GOS to yield final concentrations of up to 48 and 76 mM of raffinose and stachyose, respectively. These were the concentrations that were then used for further studies. Given that legumes contain around 5 % (w/w) α-GOS (soybeans 5.0 %, lentils 5.5 %, beans 3.2 % and chick-peas 7.5 %), the amount of α-GOS administered to rats corresponded to an intake of about 1.5 g of legumes per rat. When Cb rats received this enriched soy milk, they excreted H_2 _in expired air and flatus at up to 500 μmol/100 g metabolic weight as shown in Fig. 1. In contrast, when rats received only peptone water, only a slight H_2 _emission, probably corresponding to background dietary fermentation, was observed. These observations suggest that H_2 _emission by rats receiving enriched soy milk is linked to their consumption of α-GOS. This was confirmed by preparing soy milk in which α-GOS were degraded using an exogenous α-Gal prior to administration. In this case, a significantly lower (*P *= 0.003) H_2 _excretion was observed than when rats received enriched soy milk, indicating that the high H_2 _excretion found with the latter treatment was due to the presence of the α-GOS raffinose and stachyose from soy milk. Nevertheless, α-GOS-free soy milk induced a significantly higher H_2 _emission than peptone water (*P* < 0.05). This was probably related to the presence of non-digestible but fermentable sugars, other than α-GOS, that remained in the soy milk preparation after the α-Gal treatment [16].

### α-Gal-producing *L. fermentum *CRL722 is able to remove flatus-generating α-GOS both in soy milk and in the gastrointestinal tract of rats monoassociated with *C. butyricum *DSM10702

**Figure 2:**
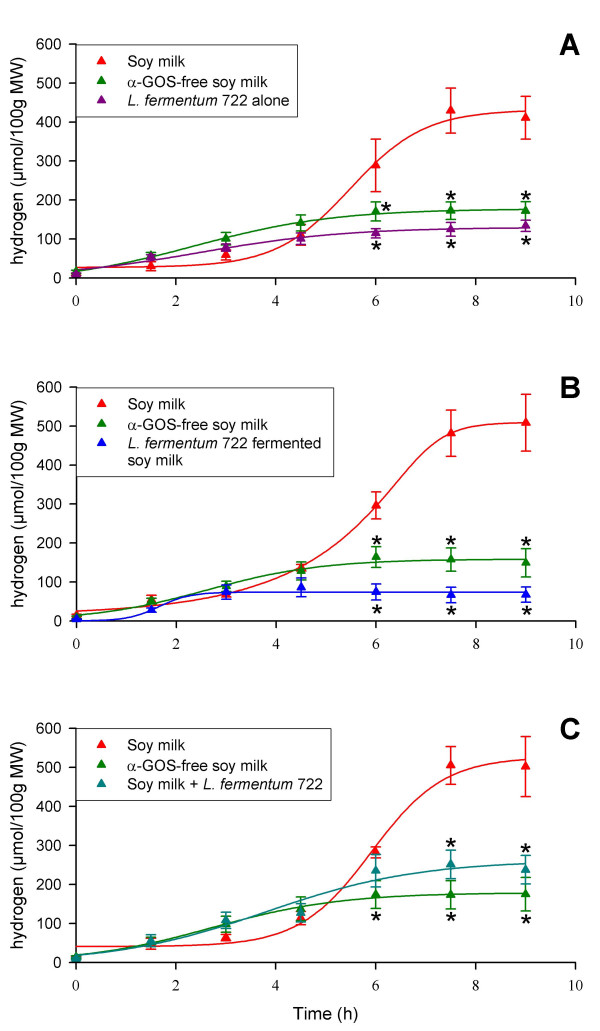
**Ability of *L. fermentum *CRL722 to reduce α-GOS-induced H_2 _excretion in gnotobiotic rats monoassociated with *C. butyricum *DSM10702**. Same legend as Fig. 1, except for the number of rats (*n *= 4). A, Cb-A rats; B, Cb-B rats; C, Cb-C rats (see Fig 5).

**Figure 5:**
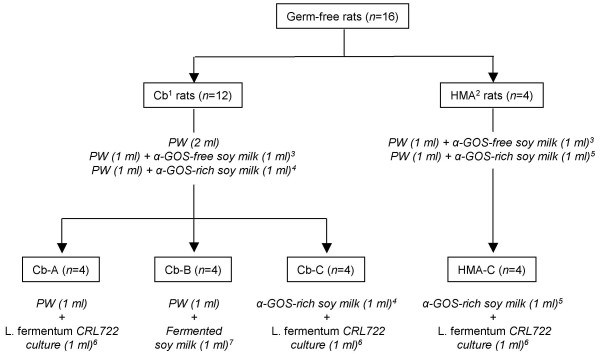
**Experimental design of the rat trials**. Treatments applied to rats are indicated in italics and numbers of treated rats in brackets. For more details, see Material and Methods section. PW, peptone water. ^1^, Rats associated with *C. butyricum *DSM10702; ^2^, rats associated with a human faecal microbiota; ^3^, α-GOS-enriched soy milk (see ^4 ^and ^5^) was added with a 5 % (v/v) coffee bean α-Gal solution at 1 IU/ml and was incubated for 12 h at 37°C; ^4^, the concentrations of raffinose and stachyose in the α-GOS-enriched soy milk administered to Cb rats were 48 and 76 mM, respectively; ^5^, the concentrations of raffinose and stachyose in the α-GOS-enriched soy milk administered to HMA rats were 144 and 228 mM, respectively; ^6^, *L. fermentum *cultures were at 4 × 10^9 ^CFU/ml; ^7^, α-GOS-enriched soy milk (see ^4^) was added with a 2 % (v/v) inoculum of a 4 × 10^9 ^CFU/ml *L. fermentum *CRL722 suspension and was allowed to ferment for 16 h at 37°C.

Prior to assessing the potential of *L. fermentum *CRL722 to allow the digestion of α-GOS in soy milk and in the rat gastrointestinal tract, the effect of the strain alone was assessed in a group of 4 Cb rats taken from the group of 12 rats tested above (Cb-A rats, Figs. 2 and 5). The administration of *L. fermentum *CRL722 to these rats had no effect on H_2 _excretion (Fig. 2A). H_2 _emission following this treatment was close to that observed previously in the same rats receiving α-GOS-free soy milk. *L. fermentum *CRL722 was used to ferment enriched soy milk whose raffinose and stachyose content was monitored during the fermentation process. This allowed the degradation of about 90 % of raffinose and stachyose (data not shown). The resulting product administered to Cb-B rats induced a slight H_2 _emission that was significantly lower (*P *= 0.004) than that observed with α-GOS-rich soy milk (Fig. 2B). This suggests that the degradation of α-GOS by *L. fermentum *CRL722 upon the fermentation of soy milk, prevented H_2 _excretion in rats thus confirming that this excretion was due to α-GOS fermentation in rats. We then tested the potential of *L. fermentum *CRL722 as a vehicle for α-Gal in the gastrointestinal tract of rats. To do this, α-GOS-enriched soy milk and a bacterial suspension of *L. fermentum *CRL722 were simultaneously given to the Cb-C rats. These rats showed an H_2 _excretion that was 50 % lower than when they received soy milk only (*P *= 0.019), and that was similar to values obtained when they were given α-GOS-free soy milk (*P *> 0.05; Fig. 2C). Analysis of bacterial populations from the faeces up to 72 h after the experiment revealed that *L. fermentum *CRL722 was present and stable at 10^6 ^CFU per g of faeces. This indicates that *L. fermentum *CRL722 was able to persist in the rats monoassociated with *C. butyricum *DSM10702. Therefore, the observed α-GOS digestion activity was possibly caused by (i) delivery of α-Gal to the gastrointestinal tract following *L. fermentum *lysis, or (ii) consumption of α-GOS by a viable and colonizing *L. fermentum *population.

### α-Gal-producing *L. fermentum *CRL722 also exerts a digestive activity in human microbiota-associated rats

**Figure 3:**
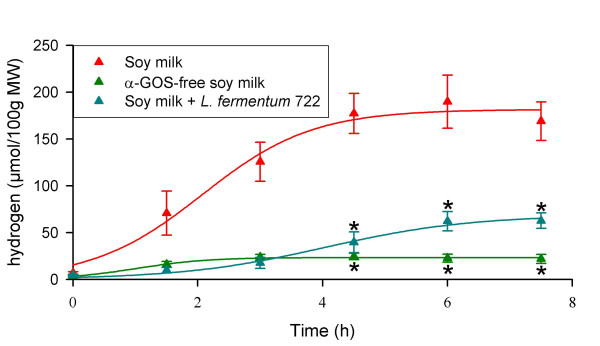
**Ability of *L. fermentum *CRL722 to reduce α-GOS-induced H_2 _excretion in human microbiota-associated (HMA) rats**. Same legend as Fig. 1, except for the number of rats (*n *= 4).

In order to more closely mimic conditions in the human gastrointestinal tract, the animal model described above was modified so that the rat gastrointestinal tract was colonized by a human faecal microbiota. In this complex microbial digestive environment, rats fed with the α-GOS-rich soy milk used for Cb rats did not excrete significantly more H_2 _than rats given α-GOS-free soy milk (data not shown). This may have been the result of either a low level of gas-producing bacteria in the human microbiota used as an inoculum, or, more probably, from the activity of H_2_-consuming microorganisms [17]. Trials with 1 ml soy milk with concentrations of raffinose and stachyose that were artificially increased 2- or 3-fold higher were performed. These trials showed that human microbiota-associated (HMA)-rats administered with soy milk whose α-GOS concentration was enhanced 3-fold, *i.e*. 144 and 228 mM raffinose and stachyose, respectively, exhibited H_2 _excretion levels of the same order of magnitude as those observed in Cb rats (Fig. 3). Under these new conditions, the activity of *L. fermentum *CRL722 was again assessed by coadministration of the strain with the α-GOS-rich soy milk. This experiment showed that H_2 _emission was reduced by about 70 % compared with the α-GOS-rich soy milk treatment (*P* = 0.004, Fig. 3), and this reduction attained statistical significance 3 h after feeding (*P *= 0.012). Monitoring of the *L. fermentum *CRL722 population present in faecal samples showed that the population decreased 100-fold each day, indicating that, in contrast with the situation observed in Cb rats, this strain was unable to colonize the gastrointestinal tract of HMA rats.

## Discussion

The primary aim of this work was to assess the potential of LAB to facilitate the digestion of soy α-GOS through their α-Gal activity. In this context, we have previously characterized α-Gals from various lactobacilli, and one from *L. fermentum *CRL722 appeared to be the most active [10]. The present work focused on assessing the ability of the α-Gal producing *L. fermentum *CRL722 strain to improve the digestibility of soy α-GOS *via *its α-Gal activity expressed either during soy milk fermentation or in the gastrointestinal tract of rats. Using an animal model consisting of gnotobiotic rats monoassociated with an H_2_-producing *C. butyricum *strain, we first showed that depletion of α-GOS from soy milk using *L. fermentum *CRL722 as a starter greatly decreased sugar fermentation in the gastrointestinal tract of rats as shown by an important reduction in H_2 _excretion. We further showed that *L. fermentum *CRL722, when coadministered with soy milk rich in α-GOS, greatly reduced H_2 _excretion derived from α-GOS fermentation. This suggests that *L. fermentum *CRL722 α-Gal is active and available in the intestine to degrade α-GOS. In our animal model consisting of gnotobiotic rats monoassociated with an H_2_-producing *C. butyricum *strain, we observed that *L. fermentum *CRL722 was able to colonize the gastrointestinal tract. This means that at least a fraction of the *L. fermentum *CRL722 inoculum survived the conditions encountered in the upper part of the gastro-intestinal tract and found an appropriate environment to multiply in the large intestine, despite the presence of a resident species. As a consequence, the observed reduction in gas production may have been caused either by the metabolic activity of *L. fermentum *CRL722 in the small intestine or as a result of the strain colonizing the large intestine and competing there with *C. butyricum *for α-GOS utilization. To assess which of these mechanisms applied, we went on to use another animal model consisting of rats harbouring a human faecal microbiota. This latter was expected to form a natural defence barrier [18], preventing *L. fermentum *CRL722 to settle in the large intestine. Using this system, the α-GOS amount previously administered to Cb rats did induce a lower H_2 _emission than that monitored in Cb rats. This may result either from a poor level of gas producing bacteria in the used human microbiota, or, most likely, from the activity of H_2_-consuming microorganisms such as methanogenic archeae, sulfatoreducing or reductive acetogenic bacteria. Therefore, the α-GOS amount administered to HMA rats was increased 3 fold. This corresponds to an average intake of 4.5 g of legumes which is a physiologically acceptable dose for rats. Under these conditions, *L. fermentum *CRL722 still improved α-GOS digestibility although, as expected, it was unable to colonize the large intestine. This strongly suggests that the enzyme activity occurs in the small intestinal compartment. In further preliminary experiments to distinguish between *L. fermentum *CRL722 α-GOS utilization and delivery of α-Gal activity by *L. fermentum *CRL722, we used another α-Gal^+ ^*L. fermentum *strain that is deficient in α-GOS transport. Interestingly, this *L. fermentum *strain exhibited an *in vivo *effect similar to that of *L. fermentum *CRL722, suggesting that the latter strain acts through delivery of α-Gal to the small intestine rather than *via *α-GOS utilization (data not shown). This observation is consistent with a previous study in which we found that the majority of *L. fermentum *CRL722 cells did not survive the duodenal portion of the small intestine, as has been found for another LAB, *Lactococcus lactis *[15,19]. In this latter work, a significant, although short-lived, α-Gal activity was found in the duodenum of rats fed *L. fermentum *CRL722. It appears that this short life of α-Gal is sufficient to allow digestion of the α-GOS present in the diet, as shown by the present work.

The model presented here should allow further elucidation of both the mechanisms and the parameters that govern the expression of LAB enzymatic activities *in vivo*. Few other studies have been performed in this area. *Lactococcus lactis *strains genetically engineered to produce lipase have been shown to be capable of alleviating a lipase pancreatic insufficiency caused by ligation of the pancreatic duct in pigs [20]. Other studies have suggested that LAB improve lactose digestion of dairy products in lactase-deficient individuals by delivery of β-galactosidase to the gastrointestinal tract [21]. However, a recent systematic review stressed the variability found in these studies and the difficulty in drawing definitive conclusions on the positive effect of LAB in lactase supplementation [22]. The α-Gal host deficiency retained in the present model system is, in contrast to lipase and lactase deficiencies, constitutive in mammals; this model should therefore reduce inter-individual variation. Furthermore, the animal model described here makes it possible to perform quick analyses. This will allow the effect of different parameters such as the influence of the LAB vehicle (susceptibility to lysis, colonizing ability, etc.) and of the mode of administration of LAB to be studied.

## Conclusion

This study showed that a given host enzyme deficiency can be supplemented by orally administered LAB that produce this enzyme at high level. This type of work opens up the possibility of using LAB vehicles for delivery of other digestive enzymes to the digestive tract. LABs have great potential in this field. First, since these bacteria have long been ingested by man in fermented products, the mucosal digestive immune system has developed a tolerance to these bacteria, some of which are commensal [23]. Furthermore, LABs form a heterogeneous family of bacteria originating from various ecological niches. These bacteria therefore have a diverse range of enzymatic activities required for their adaptation to those niches [24]. Finally, their designation as GRAS (Generally Recognized As Safe) by the US Food and Drug Administration, positions them as particularly well-suited organisms for this type of application.

## Methods

### Chemicals and culture media

Soy milk was kindly provided by Lallemand Inc. (Montreal, Canada). Raffinose, stachyose, *p*-nitrophenyl-α-D-galactopyranoside (pNPG) and α-Gal from green coffee beans were purchased from Sigma-Aldrich (Saint-Quentin-Fallavier, France). 5-Bromo-4-chloro-3-indolyl α-D-galactopyranoside (X-α-Gal) was from Research Organics (Cleveland, OH). Man Rogosa Sharp (MRS) medium was prepared as described elsewhere [25], Brain Heart Infusion (BHI) medium was purchased from Difco (Detroit, MI) and peptone water contained 1 g/l peptone (Difco).

### Bacterial strains

*Lactobacillus fermentum *CRL722 was obtained from the Culture Collection of the Centro de Referencia para Lactobacilos (CERELA, Tucuman, Argentina) and has been characterized elsewhere [10,11,14]. A spontaneous rifampicin-resistant (Rif^R^) derivative was checked for the α-Gal^+ ^phenotype, using the chromogenic substrate X-α-Gal. Briefly, bacteria were grown on MRS agar plates containing 0.5 % (w/v) glucose and spread with 50 μl of a 20 g/l X-α-Gal solution using glass beads. Quantification of α-Gal activity was performed according to a previously described method [26], using pNPG as a substrate. No significant differences in α-Gal activity could be found between the Rif^R ^derivative and the parental strain. *L. fermentum *CRL722 and its Rif^R ^derivative were routinely grown at 37°C under anaerobiosis in MRS medium, supplemented with 100 μg/ml rifampicin when required. *Clostridium butyricum *DSM10702 was obtained from the German Collection of Microorganisms and Cell Cultures (DSMZ, Braunschweig, Germany); it originates from the intestine of swine and is the type strain of the species. It was chosen because of its ability to produce large amounts of gas, namely hydrogen and carbon dioxide, from raffinose and stachyose fermentation [27]. *C. butyricum *DSM10702 was routinely grown in BHI medium at 37°C under anaerobiosis. All strains were stored at -80°C in 20 % glycerol/80 % culture medium (v/v). The human faecal microbiota originated from stools provided by a 37-year-old Caucasian woman with Western dietary habits who had not taken any laxative or antibiotic during the preceeding three months.

### Animals

Germ-free 10–12 week old male F344 rats (mean weight 266 g (SD 19 g)) were obtained from the Germ-Free Rodent Breeding Facilities of UR910 – Unit of Ecology and Physiology of the Digestive Tract (INRA, Jouy-en-Josas, France). They were randomly separated into two groups and housed in a series of Plexiglas^® ^isolators fitted with a double-door sealed transfer system (Ingénia, Vitry-sur-Seine, France). Throughout the study, rats were kept in pairs in standard macrolon cages with sterile wood shavings as bedding. They were given free access to autoclaved tap water and to a pelleted semi-synthetic diet (Scientific Animal Food and Engineering, Augy, France) sterilized by γ-irradiation at 45 kGy (IBA Mediris, Fleurus, Belgium) [28]. Isolators were maintained under controlled conditions of light (07.00–19.00 h), temperature (20–22°C) and humidity (45–55 %).

### Respiratory chambers and H_2 _excretion analysis

**Figure 4:**
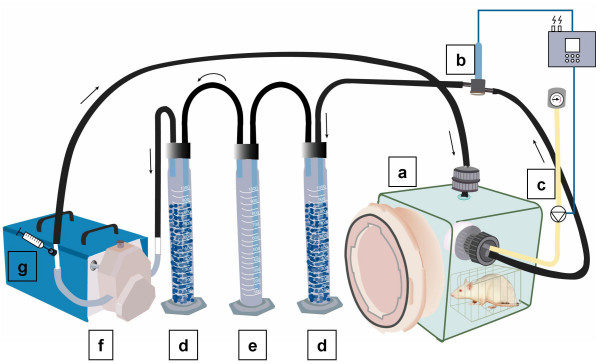
**Typical respiratory chamber used to monitor H_2 _excretion in rats**. a, respiratory chamber fitted with a rapid transfer system allowing connection to isolators housing gnotobiotic rats; b, oxygen probe; c, oxygen inlet controlled with a magnetic valve; d, silica gel-containing cylinder to trap moisture; e, KOH-containing cylinder to trap CO_2_; f, peristaltic pump; g, sampling port with syringe. Arrows indicate the direction of air flow through the system.

Respiratory chambers used in the present study have been previously described in detail, and have been routinely used for measuring H_2 _and CH_4 _excretion in gnotobiotic animals exposed to various dietary regimens [29-32]. They are shown in Fig. 4. Briefly, they consisted of sterile 32-litre cubic boxes made of thick polyvinyl chloride and equipped with a polyethylene lid fitting the double-door sealed transfer system of the isolators, to allow rapid transfer of animals without breaking containment. Within the chamber, the rat was placed in a macrolon cage with a wire mesh in the bottom and a water-containing plastic bottle on the wire lid. Both chamber and cage were transparent so that the animal could be observed. During the test period, chambers were operated in closed-circuit mode. Air circulation was maintained by a gas-tight and adjustable peristaltic pump (Masterflex, Cole-Parmer Instrument Co., Vernon Hills, IL) at a flow rate of 120 litre/h. Thus, air circulated continuously through Tygon tubing (inner diameter 9.0 mm), passing through sterile paper filters (Camfil SAS, La Garenne Colombes, France) on entering and leaving the chamber. Moisture and CO_2 _were removed by including 1-litre cylinders filled with silica gel and 7 M-potassium hydroxide, respectively, in the circuit. An O_2_-sensor (Mettler Toledo Analyse Industrielle, Paris, France) was connected to the air circuit. As O_2 _concentrations decreased due to rat respiration, the sensor triggered an electromagnetic valve that allowed gas entry from a cylinder of compressed O_2 _(Air Liquide, Paris, France). In this system, H_2 _and CH_4 _produced by gastrointestinal fermentation and excreted *via *breath and flatulence accumulated. Air samples were taken at intervals through a sampling-port, using a gas tight 50-ml syringe (Terumo Europe, Leuven, Belgium). H_2 _and CH_4 _concentrations were immediately determined by GC using a QuinTron Model DP MicroLyzer (QuinTron Instrument Company, Milwaukee, WI) with a solid-state sensor detector (sensitivity 1 ppm, accuracy 2 ppm, linear range 2–150 ppm). The QuinTron MicroLyzer was operated according to the manufacturer's instructions: the injection port was fitted with a tube containing a Drierite desiccant to allow for drying of the air samples; injection volume was 20 ml and medical-grade air was used as the carrier gas (23 ml/min); external calibration was carried out twice daily with a QuinGas™ reference standard (QT07021-G, QuinTron Instrument Company).

### Preparation of the *L. fermentum *CRL722 inoculum and the α-GOS rich- and α-GOS-free soy milk

In order to selectively quantify *L. fermentum *CRL722 in the rat faeces, the spontaneous Rif^R ^derivative was used in the animal trials. The Rif^R ^strain was grown overnight in MRS broth. The following morning, bacterial cells were pelleted and suspended in peptone water at a concentration of 4 × 10^9 ^CFU/ml for immediate use. Soy milk was sterilized by autoclaving (10 min, 110°C) and enriched with aqueous sterile stock solutions of raffinose and stachyose. Final concentrations of raffinose and stachyose in soy milk were 48 and 76 mM, respectively, for rats monoassociated with *C. butyricum *DSM10702 and 144 and 228 mM, respectively, for human microbiota-associated rats. The α-GOS-free soy milk was prepared by incubating the α-GOS-enriched soy milk with coffee bean α-Gal (5 % of a 1 IU/ml stock solution, v/v) at 37°C for 12 h. The enzyme was then inactivated by heat treatment (10 min, 100°C) and total degradation of raffinose and stachyose was confirmed by HPLC (Waters 625LC system, Mildford, MA) using a Bio-Rad Aminex HPX-87H column (Bio-Rad Labs, Hercules, CA). Elution was performed at 25°C with an isocratic flow of 0.4 ml/min 0.005 N-phosphoric acid. Eluted sugars were detected by differential refractometry (Waters 410). Sugar quantification was performed using the external standard method [33]. Both soy milk preparations were stored at -20°C until use.

### Experimental design of the rat trials

All procedures were carried out in accordance with the European guidelines for the care and use of laboratory animals. Animals of one group (*n *= 12) were inoculated intragastrically with 1 ml of an overnight culture of *C. butyricum *DSM10702 (10^10 ^CFU/ml) using a stainless-steel stomach tube; they were subsequently designated as Cb (*C. butyricum*) rats. Animals belonging to the other group (*n *= 4) were colonized with a faecal suspension made from the stools of the human donor, as described previously [34]. Briefly, fresh stools were introduced into an anaerobic glove box and dispersed in BHI broth (1 %, w/v); aliquots of the suspension were subsequently transferred to the isolators and given to the rats with a stomach tube (1 ml/rat); these rats were designated as HMA (human microbiota-associated) rats. The experiment began after a 3-wk acclimatization period to the new bacterial status. First, fresh faecal pellets were collected from each rat to confirm the microbial colonization of the gastrointestinal tract. Serial 10-fold suspensions, prepared from faecal samples of Cb rats, were spread onto BHI agar plates which were then incubated under anoxic conditions at 37°C for 48 h prior to colony counting. In HMA rats, the microbiological abundance and diversity of the faeces was assessed by light microscopic examination. The Cb rats were then randomly allocated to three groups (*n *= 4), namely Cb-A, Cb-B and Cb-C (Fig. 5). In all groups, each rat was successively fed orally with (i) peptone water to determine the basal level of H_2 _excretion, (ii) the α-GOS-free soy milk as a negative control, and (iii) the α-GOS-rich soy milk as a positive control. The fourth treatment differed according to the group: the Cb-A rats were fed with *L. fermentum *CRL722 alone to assess whether the bacterium *per se*, affected H_2 _excretion (strain control); the Cb-B rats received the α-GOS-rich soy milk previously incubated with the *L. fermentum *inoculum (2 %, v/v) for 16 h at 37°C under anaerobiosis; finally, the Cb-C rats received the α-GOS-rich soy milk along with the *L. fermentum *inoculum. In the case of the HMA rats, they received successively (i) the α-GOS-free soy milk (negative control), (ii) the α-GOS-rich soy milk (positive control), and (iii) the combination of the α-GOS-rich soy milk and the *L. fermentum *inoculum. In both Cb- and HMA groups, a one-week wash out period was allowed between each treatment. The trials were performed as follows. After overnight deprivation of food, the rats were fed with a 2 ml-mixture, the composition of which is detailed in Fig. 5, using a stainless-steel stomach tube. Immediately after feeding, each rat was transferred to a respiratory chamber where it remained for up to 9 h, and air samples were taken in duplicate every 90 min for H_2 _analysis. Rats were thereafter returned to their original isolators and fresh faecal pellets were collected at intervals for 72 h to enumerate the *L. fermentum *population.

### Enumeration of *L. fermentum *CRL722 in rat faeces

Freshly-collected faecal pellets were thoroughly homogenized in peptone water and serial 10-fold dilutions were prepared. These were spread onto MRS agar plates that contained rifampicin at 100 μg/ml, and plates were incubated under anoxia at 37°C for 48 h prior to colony counting. Under these conditions, only the Rif^R ^*L. fermentum *derivative could grow while both *C*. *butyricum *and the microbiota of human origin were inhibited. Bacterial counts were expressed as CFU/g of faeces.

### Calculations and statistical analyses

All data are expressed as mean values of H_2 _excretion per 100 g of rat metabolic weight (MW; live weight to the 0.75 power) and standard errors of the mean. In each group of rats, the effect of the treatments on H_2 _excretion was assessed using ANOVA for repeated measurements. When ANOVA indicated significant differences, treatments were compared using the Student-Newman-Keuls multiple comparison test. Statistical significance was set at *P *< 0.05. Calculations were performed using the SigmaStat software package (Systat Software Inc., Richmond, CA).

## Abbreviations

α-Gal: α-galactosidase; α-GOS: α-galacto-oligosaccharides; BHI: Brain heart infusion; Cb: *Clostridium butyricum*; CFU: Colony-forming unit; HMA: Human microbiota-associated; LAB: Lactic acid bacteria; MRS: Man Rogosa Sharp; MW: Metabolic weight; pNPG: *p*-nitrophenyl-α-D-galactopyranoside; X-α-Gal: 5-Bromo-4-chloro-3-indolyl α-D-galactopyranoside.

## Authors' contributions

JGL, FLC, MB, and TG carried out the microbiological work and the animal studies. GSG, FS, and JCP conceived the study. JGL, SR, and JCP designed the experiments. FLC, VJ, and SR performed the statistical analyses and prepared the figures. JGL and JCP wrote the draft of the manuscript. GSG, FS, SR, and VJ revised it for significant intellectual content. All authors read and approved the final version of the manuscript.
